# Nurses’ perceptions about the use of virtual reality simulation to develop competencies in managing violent and threatening behaviours: a qualitative study

**DOI:** 10.1186/s12912-026-04296-6

**Published:** 2026-01-10

**Authors:** Marianne Hoff, Jørghild K. Jensen

**Affiliations:** 1 Faculty of Health Sciences, VID Specialized University, Oslo, Norway; 2 Diakonhjemmet Hospital, VID Specialized University, Oslo, Norway

**Keywords:** Virtual reality simulation, Competence development, Nurses, Violent and threatening behaviour

## Abstract

**Background:**

Globally, there has been an increase in incidents of acting out in general hospital wards. Nurses’ competencies can play an important role in addressing this issue, while at the same time, the use of virtual reality simulation to support knowledge and skills development is rapidly developing in the healthcare field. The purpose of this study was to explore general hospital ward nurses’ perceptions about the use of virtual reality simulation to enhance their competencies in managing violent and threatening behaviours.

**Methods:**

A qualitative interpretive study design through focus groups with twenty-five general hospital ward nurses who had participated in virtual reality simulation was conducted. A semi-structured interview guide facilitated the two focus group interviews. Data were analysed thematically using Braun and Clarke’s six-phase framework to understand and interpret the nurses’ perceptions regarding competency development.

**Results:**

Three main themes emerged: (1) the development of competencies related to relational communication and risk assessment; (2) the value of virtual reality simulation as a complement to other educational methods for competency development; and (3) nurses’ perceptions of the barriers and facilitators associated with the use of virtual reality simulation for competency enhancement.

**Conclusions:**

The results of this study support the use of virtual reality simulation to enhance competencies in managing violent and threatening behaviours, although it is important that virtual reality simulation supplement other pedagogical learning methods. Virtual reality simulation can be used to enhance the learning experience and prepare healthcare workers for their roles. A greater understanding of using virtual reality simulation in healthcare settings is needed to utilize this technology effectively for competency development.

**Supplementary Information:**

The online version contains supplementary material available at 10.1186/s12912-026-04296-6.

## Background

Numerous studies have documented a global rise in incidents of acting out, violence, and threats among people receiving treatment in general hospital wards [1–4]. Nurses are the primary victims of such behaviour [1–3, 5], and nurses’ competencies can play an important role in managing these challenges. Ensuring workforce competence is vital for maintaining safety. Simulation-based training is an effective method for enhancing competencies because aspects of the real world can be replicated, providing a safe learning environment [6, 7]. The advancement of technology has brought about significant changes in healthcare delivery, with virtual reality (VR) emerging as a rapidly expanding tool within this field [8–10]. A scoping review highlighted the integration of VR into healthcare education as a novel and underexplored area of research [11]. These insights might be transferable to clinical practice and healthcare professionals. Further studies are needed to deepen our understanding on the use of VR simulation to enhance nurse competencies and to improve these applications. Consequently, this study aims to explore the perceptions of general hospital ward nurses regarding the use of VR simulation to enhance their competencies in managing violent and threatening behaviours.

In general hospital wards in Norway, ongoing initiatives emphasize the development of competencies among healthcare professionals, particularly in relation to managing exposure to violence and threats. Enhancing knowledge and skills through education and targeted training in prevention and response strategies is currently being prioritized. As a result, the ability of nurses to effectively address situations involving violent or threatening behaviour remains a critical concern within the broader context of hospital practices. Nurses’ ability to remain calm may influence the progression of threatening situations. Experiences of violence and threatening behaviour can lead to serious consequences, including emotional exhaustion, stress, sleep disturbances, increased sick leave, and reduced job satisfaction [12, 13]. Two recent studies have explored the use of virtual simulated learning experiences [14, 15]. Conrad et al. [15] conducted an evaluation of undergraduate nursing students’ perceptions of confidence and success in their de-escalation skills following a virtual simulation intervention. They found that virtual simulation positively affected participants’ feelings of confidence and success in these skills. Additionally, Furuheim and Lindenskov [14] investigated the use of VR simulation as a learning activity to enhance confidence and communication skills among nursing students in mental health practice. Their findings indicate that VR simulation serves as a valuable preparation and supplement for clinical practice in mental health, enabling students to acquire practical and relational competencies. A study by Lockertsen and Kjærvik from 2025 [16] examined staff experiences with VR simulation as a supplement to physical simulation for de-escalation in mental health settings. The findings indicate that VR simulation enhances situational understanding but does not sufficiently prepare participants for action readiness. Furthermore, the authors suggest that VR simulation cannot replace physical simulation in de-escalation training. Notably, none of the participants reported any negative emotional impact associated with the use of VR simulation.

Nurses’ competence is a complex concept. Competence combines and includes knowledge, performance, skills, attitudes, and values [17, 18]. Competence development is essential for healthcare services to attract and retain employees. To foster the development of employee competencies, the community’s learning environment holds considerable significance. This environment is a dynamic and complex construct shaped by individuals within a specific context [19]. A positive learning environment, characterized by an open and supporting atmosphere, enhances satisfaction, well-being, academic performance, and collaboration. In contrast, a negative learning environment may hinder participation and learning, potentially resulting in emotional exhaustion, depersonalization, and burnout [19]. Establishing a psychologically safe environment during simulation has garnered attention in recent years. Participation in simulation can evoke feelings of exposure because participants may fear revealing knowledge gaps and perceive feedback on performance as threatening. In this context, psychological safety becomes crucial. Psychological safety can be defined as a condition that allows individuals to feel safe and comfortable expressing themselves and to take interpersonal risks without fear of negative consequences [20].

There is an ongoing challenge in identifying accessible and innovative teaching and learning methods to enhance nurses’ competencies [9, 10]. Simulation-based learning offers an alternative approach and is a recognized method for competency development [6, 8–10, 21]. During simulation-based learning, the simulation methodology is followed. This methodology includes briefing, simulation, and debriefing sessions [22, 23]. Simulation starts with a briefing session that outlines participants’ expectations and may vary based on their experience and theoretical framework. In the second session, the actual simulation takes place. Finally, there is a debriefing session, which provides the opportunity for the participants to reflect on and discuss the situation and learning goals. The nature of simulation can lead participants to feel vulnerable and psychological safety is important to maintain. The objective is to create a safe and engaging environment that fosters reflection and learning, thereby enhancing the participants’ competencies. Evidence shows that essential learning occurs during the debriefing phase [23–27].

Healthcare studies indicate that VR applications are rapidly developing in the healthcare field, with the increasing usage of this innovative technology in recent years [8]. VR is a computer-generated, three-dimensional environment that allows users to interact with it, typically accessed via a display such as a head-mounted display or screens. It is divided into two categories: non-immersive VR, which uses surrounding screens, and immersive VR, which employs wearable displays to create a fully immersive experience [28]. Immersive VR has been investigated as a pedagogical tool in education and training [29–31]. A systematic literature review by Hamilton, McKechnie, Edgerton and Wilson [31] found that most studies reported significant advantages of using immersive VR in education. In contrast, Jensen and Konradsen’s [30] review highlighted the limited effectiveness of immersive VR, citing concerns about the relatively low quality of the studies included. A more recent systematic review from 2024 indicates that immersive VR positively impacts learning compared to other media types and is well-suited for environments that prioritize active learner engagement and practical application [29]. The motivation for employing immersive VR in education and training lies in its ability to expose learners to challenging situations and allow them to practice new skills to develop competencies. Using immersive VR offer advantages in achieving specific learning objectives. In summary, immersive VR shows promise as a teaching tool. Compared to traditional learning methods, VR technology provides a more immersive medium for both theoretical and clinical education [9, 10]. By utilizing a head-mounted display, VR creates a three-dimensional (3D) environment that fully immerses the user, allowing interaction within the virtual space through hand controllers [32]. This technology enables training in authentic patient scenarios and offering valuable learning experiences [8, 15, 16, 29, 31]. However, it has been argued that VR should complement, rather than replace, conventional teaching and learning methods [9, 10, 16].

It has been found that emotions can both hinder and promote learning in traditional simulation training [33]. Traditional simulation with a simulator and having observers present during the simulation, can induce negative feelings such as stress, fear, and anxiety due to performance pressure [34, 35]. Al-Ghareeb, Cooper and McKenna [34] showed that anxiety can either improve or impair clinical performance during traditional simulations. Also, in VR simulations, emotions such as fear, empathy, stress, and irritation can be triggered [36]. Research indicates that emotions influence perception, memory, attention, and the transfer of learning to new situations. Recognizing the role of emotions can deepen our understanding of their impact on teaching and learning [14, 36, 37]. However, using VR also introduces the challenge of cybersickness, which can impact the user experience, emotions, and learning [9, 11, 30]. The use of VR can combine healthcare simulation methodologies with 360-degree video scenarios to train healthcare professionals in clinical situations. In this context, psychological safety among participants during reflection may facilitate collaborative learning, promoting competency development related to the scenarios and individual practices. Reflection occurs during the debriefing sessions after the participants have engaged with the 360-degree videos of clinical situations via VR a head-mounted display.

A systematic review conducted in 2022 underscores the significance of managing violent and threatening behaviour in mental health settings [38], and these findings may be applicable to general hospital wards. VR is an emerging tool for training healthcare providers. Previous studies indicate that nursing students find simulation using VR technology beneficial for their learning [7, 14, 15, 36], suggesting that similar perceptions may extend to registered nurses. Consequently, this study’s aim is to explore how general hospital ward nurses perceive the use of VR simulation for enhancing their competencies in managing violent and threatening behaviours.

## The initiative before the study started

Before our study commenced, a Norwegian hospital had initiated efforts to implement VR simulation in the training of nurses to enhance their competencies in managing violent and threatening behaviours. Two researchers (MH and JKJ) were invited to explore the perceptions of nurses regarding the VR simulation. The hospital and the university, to which the researchers belong, have a history of collaboration and interaction in research and professional development. The training was developed in the autumn of 2021 by a group of managers and professionals at the hospital. The training lasted for three hours and included the theoretical foundation regarding violent and threatening behaviour. This included a short web-based introduction followed by a presentation of the guidelines, how to complete a risk assessment, how to prevent harm, advice in the event of threatening behaviour, and the implementation of measures in the case of incidents. Following this session, the nurses participated in the simulation using an Oculus Quest 2 VR head-mounted display and observing one single scenario. In the introduction to the VR simulation, the briefing emphasized the theme of the 360-degree video to be observed, the learning objectives, and information regarding the VR head-mounted display. During the simulation, the participants observed a recording of a role play made by professionals within the field of study. During the role play, a patient admitted to a general hospital ward became threatening towards the nurses. The recording of the role play demonstrated a good example of managing a threatening patient, while also highlighting certain areas for improvement, such as the nurses having scissors attached to their uniform. The participants gained a shared experience by viewing the same 360-degree video. The nurses participated individually but were in the same room during the simulation. The scenario lasted for 10 min. The VR simulation debriefing was facilitated by a facilitator to enhance learning and competence development.

## Methods

### Study design

This study adopted an interpretive qualitative approach to gain a deeper understanding of the participants’ perceptions of the use of VR simulation in relation to competency development for managing violent and threatening behaviour. A qualitative interpretive research design was chosen due to its suitability for capturing participants experiences with VR simulation, involving the analysis of two focus groups conducted with ward nurses who had participated in VR simulation training on managing violent and threatening behaviour.

### Participants

The nurses who had attended the course described in the previous section were asked by the course instructor to participate in the study. Twenty-five nurses (three male, 22 female) from hospital medical wards participated in the study. Their ages ranged from 23 to 45 years, and their levels of experience varied. Some were newly graduated, while others had several years of experience. All the participants agreed to take part in the study.

### Data collection

Data collection involved two focus group interviews with ward nurses participated in VR simulation. 12 nurses participated in the first focus group interview and 13 nurses in the second. A semi-structured interview guide was developed by the researchers, informed by the literature and the research aim. The interview guide was specifically developed for this study and can be found in the supplementary file. The guide included various questions on how the nurses perceived the use of VR, aiming to cover the study’s main purpose. For consistency, the two focus groups were moderated by the same researchers. The focus group interview approach was chosen to capture collective engagement after the nurses had jointly completed the VR simulation. At the same time, the focus group interview method triggers collective reflection within the group based on the participants’ statements. The interviews were conducted in designated rooms with the participants, and the two researchers present (with first author MH as the leader and second author JKJ as the moderator). The interviews were digitally recorded and transcribed verbatim by the first author. The second author made notes during the focus group interviews about the dynamics in the group and nonverbal communication, and asked follow-up questions. The duration of each interview was 50 min. The researchers are both female registered nurses and have approximately 20 years of clinical experience each as hospital nurses. One of the researchers works at the university and holds a master’s degree, while the other researcher has a PhD and works both at the university and the study hospital. The researchers had no established relationships with participants prior to study commencement, although some of the participants may have known the researchers before the study took place. This study was conducted in accordance with the Declaration of Helsinki, and approval for the study was obtained from the Norwegian Centre for Research Data (NSD) and the current hospital leadership. The participants received both written and oral information about the study, and all provided written informed consent. Participants were reminded that they could withdraw from the study at any time. All data were anonymized to protect the participants’ privacy.

### Data analysis

The data from the focus group interviews were analysed using Braun and Clarke’s [39] six phases of thematic analysis, which involves systematically identifying, organizing, and obtaining insight into patterns of meaning that appear as themes across the data. The analysis was done manually by the two authors using Microsoft Word and marking up the text file. The interview texts were first read and then reread by the researchers, and we wrote down our initial thoughts separately. Then, we discussed our first impressions before conducting further analyses. Afterwards, we coded the relevant data separately and systematically, and thematic words and terms were noted in the margins during reading. The initial codes were then discussed, and these codes were revised to create a meaningful, condensed version (see Table 1 for examples of data extraction with applied codes). The codes were divided into themes. A theme was defined as a topic that possessed a meaning related to the topic of interest. The codes and themes were derived from the data. This study employed latent coding to interpret data in depth, revealing underlying meanings and themes beyond surface-level interpretations. The initial use of semantic coding informed the analysis, demonstrating the researchers’ methodological rigor and responsiveness to the data. The participants provided no feedback on the findings or themes. The researchers discussed the appropriate terms to describe the themes. In the findings section, each theme is described briefly before one or more participant quotes are presented. The quotes were selected to illustrate some of the data informing the analysis. Additionally, they illuminate how the text informed the interpretation.

**Table 1:** Examples of data extraction with applied codes

Data extract	Applied code
Conversations after using VR are very exciting, as we have different experience and varying levels of expertise.	Debriefing and reflection are an important learning arena.
The discussion we have together strengthen our awareness of how to communicate in such situations.	Debriefing and reflection are an important learning arena.
I believe that seeing something imperfect is what you learn the most from; it’s what makes you reflect together after the simulation.	Learn to communicate. Debriefing and reflection are an important learning arena.
You have to be more involved in the simulation or the case, whereas here you are a spectator. The difference between VR and a screen or normal TV is that you become much more engaged, even if you aren’t actively participating. However, the difference from a real simulation is that you might feel more of the emotions involved and you have to respond yourself in a way.	VR simulation as a complement to other educational methodsInvolvement or a spectator.
When you simulate, you use your own expertise and relay on it, whereas here you are observing others and can more easily see what might be wrong. You might think, I wouldn’t do it that way. This applies both positively and negatively; for example, you might see something done in a way you hadn’t thought of before.	Emotions during simulation VR simulation as a complement to other educational methods Involvement or a spectator.
What is positive about VR over a simulation is that you are more willing to freely offer criticism and tips.I think it was very effective compared to video, but I believe a simulation with simulator, or a marker might be better. Still, I think it was a very good alternative to video.	VR simulation as a complement to other educational methodsVR simulation as a complement to other educational methods

### Rigor and trustworthiness

Rigor and trustworthiness of the analysis was assured by following the guidelines for thematic analysis described by Braun and Clarke [39] and by considering the four criteria established by Lincoln and Guba [40], namely credibility, transferability, dependability and confirmability. Two researchers first worked independently and then discussed their interpretation. The interpretations were discussed with a research group to ensure a reflective analysis. However, the participants did not provide feedback on the analysis. In the interviews, dialogical validation was employed continuously by further exploring participants’ statements to ensure accurate understanding. The credibility of our interpretations is supported by a thorough description of the research process, presentation of the analysis, and inclusion of quotations. To ensure the transferability and dependability of results, the participants and the context in which data were collected are described in detail. Confirmability is supported by the inclusion of numerous quotations from the transcribed material. Rigor in reporting the research was attained by applying the consolidated criteria for reporting qualitative research (COREQ) [41].

### Findings

The ward nurses participating in the study provided diverse perspectives on using VR simulation to enhance competencies, particularly in relation to managing violent and threatening behaviour. The thematic analysis revealed three themes: (1) the development of competencies related to relational communication and risk assessment; (2) the value of VR simulation as a complement to other educational methods for competency development; and (3) perceptions of the barriers and facilitators associated with the use of VR for competency enhancement.

### Theme 1: The development of competencies related to relational communication and risk assessment

The term ‘competency’ is used as a particular subset of skills for handling violent and threatening behaviour. The participants perceived that the VR simulation fostered an awareness of the existing knowledge possessed by them. A common view among them was that VR simulation developed awareness through the discussion and reflection on effective practices in the debriefing. VR simulation represented a distinct method for developing competencies in relational communication skills and risk assessment, facilitating the retention of information. The participants emphasized that the debriefing session was the most important session for developing competencies, as it allowed for discussion and reflection on effective practices during the simulation and enabled them to learn from colleagues’ experiences with violent and threatening behaviour. One participant shared: *‘People have different reflective abilities*,* so getting together and reflecting after watching a case using VR can be very useful.’* In addition, the development of competencies for managing violent and threatening behaviour was divided into how the participants reflected on their relational communication skills and the competence to make a risk assessment.

#### Relational communication skills

The participants perceived increased competence in relational communication skills. One of the most important topics discussed was how to build good relations with patients. One participant shared this in one of the focus group interviews:

*When I reflect now*,* I see that the communication was no good*,* and that the nurses did not use the time needed to build a good relationship with the patient. So*,* the next time I meet a threatening patient at work*,* this will be fresh in my memory*,* and I will make building good relations with the patient a priority. It strengthens my consciousness of how to communicate in these situations.*

The participants also pointed out the importance of good communication among themselves, not only with the patient, to build good relations. This meant that they discussed the importance of role clarification and planning before entering the patient’s room. A participant from one of the focus group interviews shared: *‘It is important that one of us takes responsibility in the situation and that we have clarified what to do before we enter the room.’* Additionally, there must be alignment between verbal and nonverbal communication to prevent threatening situations from escalating. The participants emphasized the importance of interacting calmly with the patient and ensuring that verbal messages match body language. One participant shared: *‘I have become more aware of body language; you are told that if you act like that*,* it can signal different attitudes.’*

During the interviews, several participants discussed their relational communication strategies when interacting with patients exhibiting violent and threatening behaviour. They talked about how they became more attentive to how to hold their body, such as sitting down versus standing. The participants also commented that they became aware of where and how to hold their hands. A recurrent theme was that the hands should always be visible to the patient so that misunderstandings could be avoided. Relational communication emphasizes the embodied and relational aspects of de-escalation when addressing violent and threatening behaviour. One participant shared: *‘You should show your hands at all times; do not put them in your pockets or behind your back.’* The participants also discussed the importance of their placement in the room. They would always maintain a distance from the patient. A recurring phrase was, *‘Be aware of your placement in the room. Stand by the door; do not place the patient between yourself and the door.’*

#### Competence to make a risk assessment

The participants talked extensively about conducting risk assessments of patients displaying violent and threatening behaviours. Several participants talked about preparations before entering the patient’s room, discussing the importance of preparing themselves before the patient entered the ward, and performing a risk assessment with the information in their possession. They indicated the importance of preparing themselves for what they might encounter so that they could be ready if something happened. In one of the focus group interviews, one participant shared that by doing so, *‘you have thought through the scenario in a way and are more prepared’.* Several participants talked about the importance of preparing the room before the patient’s arrival, such as removing dangerous objects that could harm either the patient or the nurse. In addition, they shared that nurses should remove sharp objects, such as scissors, from their pockets. One participant shared, *‘Yes*,* I feel that I am good at checking the room*,* but I think I have probably entered such a situation with scissors in my pocket*.’ During the focus group interviews, several of the participants suggested that new employees could benefit from training with VR and risk assessment before they started working on the ward. VR training on situations involving threatening behaviour in advance of a real incident could make it easier for nurses to conduct a risk assessment and to recall what they have learned.

### Theme 2: The value of VR simulation as a complement to other educational methods for competency development

Most of the participants interviewed appreciated the VR simulations; however, several preferred traditional simulations using simulators because they felt that they could better apply their own skills in those scenarios. Nonetheless, the participants acknowledged that VR simulation could be a valuable complement to other educational methods and that integrating VR with traditional simulations could offer a more comprehensive educational experience. One participant shared the following:

*There is something about the fact that when you simulate*,* you use your own competence*,* whereas here you are a spectator to others and can see what is wrong more easily. It becomes a little easier to see other ways of solving conflicts than what you would have done yourself. There can be such a difference. They might complement each other.*

The participants found that VR simulations in which they observed 360-degree films made it easier to reflect on different approaches to managing challenging patient situations. The immersive experience allowed them to view the scenarios from multiple angles, fostering deeper insight and understanding into various conflict resolution strategies. This broadened perspective encouraged them to consider alternative methods they might not have contemplated initially, enhancing their ability to adapt and respond effectively in real-life situations. Several participants expressed during the interviews that their stress levels were low and that it was easier to comment on and criticize the performance they had watched through a VR head-mounted display. One participant shared that:

*When you are a bystander to others*,* you may easily see what is wrong or that the performer did something that you hadn’t thought of. It was a good way to solve it. It becomes a little easier to see other ways of solving conflicts than what you would have done yourself.*

The participants pointed out that it is difficult to create an authentic situation by using a simulator as in traditional simulation when the topic is violent and threatening behaviour. The VR simulation made the situation feel more real. In addition, the participants felt that they had more control and a better overview of the situation with VR. There were some suggestions that the VR simulation method was a bit too passive. One participant shared:

*In a simulation with a simulator*,* you must be more involved*,* but by using VR you are a passive spectator. The difference from real simulation is that you get to feel more of your own emotions*,* and you must answer for yourself in a way. You don’t have to do that with VR; you are just an observer.*

Overall, the participants felt that VR simulation should be used together with traditional simulation methods; however, several participants communicated that their stress levels were lower when using VR than with other forms of simulation, which led to positive outcomes. One participant shared, *‘In a simulation*,* I am afraid of doing something wrong or being afraid that people will say*,* “What was she doing there?”’*.

### Theme 3: Perceptions of the barriers and facilitators associated with the use of VR for competency enhancement

The participants shared their perceptions of using VR, with the common view that VR facilitates competency development. Most of the participants reported a high level of engagement, characterizing VR as user-friendly, enjoyable, and memorable. Several participants noted that VR experiences are more memorable than traditional presentations and conventional teaching methods. During face-to-face education, there is a risk of being preoccupied with other things and thoughts or even looking at one’s mobile phone. The participants shared their experiences of how they forgot their surroundings, were not disturbed by others, and were less disturbed by their own thoughts while using VR. They expressed increased concentration on the immersive VR content. One participant shared:

*It is much better than watching a TV screen because you feel you are more inside the situation eh … and get it a little closer to your body*,* perhaps. It is a little more reality-related than just watching a TV.*

Another participant expressed that *‘it is easier to remember something you have watched*,* rather than something you are being told. So*,* I think in terms of using VR*,* we remember it easier’.* A recurring theme in the focus group interviews was the participants’ perceptions of VR technology as user-friendly. They highlighted its simplicity, noting that VR could be effectively applied across a wide range of topics. One participant shared: *‘I feel that VR really can be used for everything*,* regardless of whether it is a procedure or simulation. It can be used very easily.’* Overall, the participants found the use of VR to be realistic and immersive, thus enhancing their engagement in the experience. They talked about improved concentration and perceived VR as user-friendly, innovative, and interactive, thereby aiding competence development. However, some participants noted barriers to using the technology. A few of the participants experienced cybersickness during the VR simulation, with one expressing: *‘Yes*,* it was fun. But it was a bit negative how my body reacted to it. I was so sick.’* Some suggested that this discomfort may have stemmed from difficulties adjusting the VR head-mounted displays. Despite these concerns, all participants agreed that VR effectively facilitates competency development.

## Discussion

The current study aimed to explore general hospital ward nurses’ perceptions of using VR simulation to develop competencies in managing violent and threatening behaviours. The analysis resulted in three main themes: (1) the development of competencies related to relational communication and risk assessment; (2) the value of VR simulation as a complement to other educational methods for competency development; and (3) perceptions of the barriers and facilitators associated with the use of VR for competency enhancement. To interpret these findings, the results are discussed in light of three themes related to nurses’ perceptions of VR simulation in the context of utilizing VR for training and competency development.

### Perceptions of the use of VR simulation to develop competencies

In this study, participants observed a scenario using a head-mounted display. This observation can be categorized as an active learning environment due to the combination of simulation methodology, despite utilizing a passive VR simulation format where participants watched a scenario using a head-mounted display. The findings revealed that participating nurses perceived improvements in their performance and attitudes, particularly regarding relational communication skills and patient risk assessment knowledge. This is in line with the scoping review conducted by Philip and Savundranayagam [42], which found that the measurable outcomes of passive VR exhibit certain advantages, such as increasing knowledge as well as improving competency skills including communication and decision-making. Watching a scenario using a head-mounted display can provide innovative modes of learning that prioritize participant immersion. It can also be an effective training tool when the priority is to educate users on a particular disease or behaviour. However, despite passive VR’s many benefits, one drawback is the inability to interact with the virtual space. This may lead to disengagement, inattentiveness, and lack of interest [42]. Our findings support existing research on the experiences of nursing students, indicating an increase in competencies associated with simulation experiences involving VR [9, 10, 14, 36, 42].

Debriefing is a key factor during simulation exercises, and competency development occurs in the debriefing phase of the simulation-based experience [23–26]. Gardner [25] regarded debriefing as ‘the heart and soul’ of the simulation experience. This is where nurses can try to make sense of what happened during the simulation and discuss what went well and what they would change or do differently the next time. Reflection is defined as the deliberate contemplation of the significance and consequences of an action [23, 24]. This includes the assimilation of knowledge, skills, and attitudes with preexisting knowledge. Consistent with the literature, our study identified the debriefing session as essential for learning within the context of VR simulations. All the participants in our study perceived that they gained valuable insights through joint reflection and discussion on effective practices, which enabled them to learn from their colleagues’ experiences with violent and threatening behaviour. This collaborative learning process suggests that the knowledge acquired through VR simulations can be transferable to real-world practice, enhancing their ability to manage similar situations in their professional roles.

Emotional activation during VR simulation may impact competency development. Emotions are a central part of learning and can either promote or hinder the learning process [33]. Madsgaard and Svellingen [20] emphasize that establishing psychological safety in simulation is important due to the potential for participants to feel anxious and stressed. Psychological safety in simulation is more nuanced than in traditional work environments in which the concept originated and is not merely about eliminating all discomfort during learning situations. Additionally, psychological safety is necessary for a challenging learning environment in which participants feel safe enough to risk failure and be exposed to dilemmas, thereby facilitating learning opportunities underscores the relevance and necessity of psychological safety, highlighting that it is a key premise for learning. Prior studies have suggested that traditional simulation methods can be a demanding form of learning and may cause negative emotions, such as stress, fear, and anxiety related to performance pressure [33–35]. Our study showed that when participating in the VR simulation, the nurses were calm, their stress levels were low, and they got an overview of the situation. Lower stress levels in learning situations can make nurses more receptive to learning [33]. In our study, the participants were engaged in observational learning by viewing a 360-degree video without active involvement, which differed from the participatory nature of traditional simulations. Nevertheless, the act of observing a VR scenario can elicit significant emotional responses [36]. For instance, witnessing a threatening patient in a VR setting may evoke discomfort in the observer, as the immersive qualities of VR foster a sense of presence within the scenario. This virtual engagement can prompt authentic emotional reactions, allowing viewers to empathize with the presented situation and reflect on their emotional responses despite their passive role. The participants in our study highlighted that the VR simulations significantly influenced their emotional states while observing the scenario through the head-mounted displays. Research supports the notion that all sensory reactions may contribute positively to the learning process [33, 36], while exposure to emotionally activating stimuli can enhance engagement [36]. Consequently, negative emotions, such as stress, fear, and anxiety, may also play an important role in the learning experience.

### Perceptions of how VR simulations can prepare novice nurses to manage violent and threatening behaviour

The nurses in our study perceived that the VR simulation set the right conditions to develop competencies to be applied in clinical practice. Furthermore, the participants indicated that new employees could benefit from receiving VR simulation training before they start working in a hospital ward. This is consistent with the scoping review by Philip and Savundranayagam [42], who found that VR was beneficial when delivering content to novice learners. VR-based training in scenarios involving violent and threatening behaviour prior to encountering real-life situations may equip nurses with essential coping strategies while ensuring the safety of both nurses and patients. This perspective is supported by research conducted by Chang and Lai [8], which illustrated that VR environments that mitigate patient risks can provide learners with valuable learning experiences. Additionally, VR can serve as a tool for reinforcing previously acquired knowledge, thereby enhancing nurses’ preparedness when they begin their roles in general hospital wards. Similar findings have been found in other studies [10, 14, 15, 29, 31] that indicate that VR has proven beneficial in education, training and preparing nursing students for their clinical placements. Collectively, these studies underscore the value of VR simulation in fostering readiness among novice nurses to confront potentially volatile situations.

### VR simulation as a complement to other educational approaches

While the participants in our study expressed perceptions of competency development using VR simulation, our findings indicate that reliance solely on VR training is insufficient to manage violent and threatening behaviour. VR has been found to be antisocial and isolating, and it could negatively impact human interactions, nursing values, and caring and compassion, and could potentially impact on nurses’ competency development [9]. Our study showed that VR simulation can be effectively used alongside other teaching methods, such as traditional simulations using simulators, to complement each other in developing nurses’ competencies. This is consistent with previous research indicating that VR simulation is perceived as a valuable supplement to physical simulation [16]. By integrating VR’s immersive observational experiences with the active participation required in traditional simulations, nurses can benefit from a comprehensive learning approach that enhances both their practical skills and reflective capabilities. This combination allows them to explore various strategies and perspectives, ultimately fostering a more holistic understanding and application of their professional expertise. This is in line with Shorey and Ng [7] systematic review, which suggested that VR may be used as an alternative and complementary teaching method in teaching theoretical knowledge in nursing. In a review study [43], it was found that VR was suitable for supplementing conventional teaching methods rather than being used as a standalone approach. In summary, our findings of using VR as a supplement to other educational approaches to develop nurses’ competencies are consistent with the literature. VR has been recognized as a promising pedagogical tool that may enhance learning [11, 14, 15, 29, 31, 36]. The participants in our study reported that VR simulation served as a facilitator for reflective practice, enabling them to enhance their relational communication skills, conduct risk assessments, and become more conscious of their body language during patient interactions. Reflections on skills in dealing with threatening behaviour during VR simulations can be transferred, and it can be assumed that nurses become more attentive to de-escalation techniques when faced with aggressive behaviour in practice. Furthermore, the nurses in our study described the VR head-mounted displays as user-friendly and both enjoyable and memorable to use. They reported heightened concentration and focus while using the technology, characterizing it as innovative and interactive. These perceptions align with similar findings that noted positive experiences among staff in mental health department and nursing students utilizing VR [10, 16, 29]. However, some of the participants in our study experienced symptoms of cybersickness during the VR simulation, which impeded their ability to maintain attention and concentration, potentially impacting their overall learning experience. These results reflect similar findings that reported instances of nausea among nursing students [10, 11, 44]. Such symptoms may limit the applicability of VR simulation for certain users. Nevertheless, most of the participants in our study regarded the use of VR technology as an effective and advantageous learning method for competency development. They viewed VR simulation as an appropriate pedagogical strategy and a complement to other educational methods aimed at enhancing nurses’ competencies. Despite the positive perceptions reported by the participants regarding competency development and their affinity for VR technology, several questions remain unanswered. Further research is needed to elucidate the long-term impacts of VR simulation on competency development for healthcare workers. Future studies could explore alternative themes beyond violent and threatening behaviour to assess the broader utility of VR simulations in healthcare settings.

### Study limitations

Some limitations must be acknowledged in this study. The research was conducted at a single state-funded hospital in Norway, with 25 participants involved, all of whom were nurses. The primary focus was on these nurses’ perceptions of using VR simulation. The findings may not be generalizable to all healthcare settings in Norway or internationally. It is possible that some of the nurses may have felt obligated to participate in the focus group interviews despite being explicitly informed that their participation was voluntary. A key limitation of the study is the omission of the participant validation step, which is essential for enhancing the credibility of qualitative findings [45]. The participants were not provided with the transcriptions or the identified themes, nor did they offer feedback on the findings. Although the researchers were engaged in consensus discussions to strengthen the analysis. Furthermore, most participants were first-time users of VR technology, potentially leading to a novelty or ‘friendliness’ bias that could skew the results in a positive direction. To mitigate a biased interpretation of the data, the two researchers independently analysed the findings. These researchers were invited to participate in the study and had no personal stake in its outcomes. Moreover, there was no prior acquaintance between the researchers and the participants, which allowed for a more unbiased exploration of the participants’ perceptions. Another key limitation is that the focus groups reflected perceptions of competency that may not align with the observable skills demonstrated in subsequent simulations or actual patient care.

## Conclusions

This study aimed to explore general hospital ward nurses’ perceptions of using VR simulation to develop competencies in managing violent and threatening behaviours. Employing a qualitative interview method allowed for a reflexive examination of the participants’ perceptions. Our findings indicate that nurses view VR simulation as an innovative and memorable approach to competency development, particularly in relational communication skills and risk assessment. The study underscores the potential of VR simulation as a valuable complement to traditional educational methods. Overall, the participants viewed VR simulation as a valuable tool for building knowledge and facilitating reflection and discussion during debriefing. VR has been identified as a valuable tool for enhancing the learning experience and developing competencies among healthcare professionals. This study demonstrates the potential of VR simulation for implementation in general hospital wards to prepare healthcare workers for situations involving violent and threatening behaviours. Given the positive perceptions and competency advancements identified in this study, there is a compelling case for the application of VR technology across various nursing specialties. Consequently, this research may have important implications for nurses in general hospital ward settings, as well as for management and nursing educators, regarding the application of VR simulation in the preparation of healthcare workers.

## Supplementary Information

Below is the link to the electronic supplementary material.

Supplementary Material 1

Supplementary Material 2

## Data Availability

The dataset and analysis supporting the conclusions of this article is available under request to the corresponding author, without undue reservation.
