# Supplementary material for: Nurses’ perceptions about the use of virtual reality simulation to develop competencies in managing violent and threatening behaviours: a qualitative study
Source: BMC Nurs. 2026 Jan 10;25:135. doi: 10.1186/s12912-026-04296-6 (PMC12882630; doi:10.1186/s12912-026-04296-6)
Supplement: Supplementary file 1 — Supplementary Material 1 [file 12912_2026_4296_MOESM1_ESM.docx]

Semi-Structured Interview Guide

At the beginning of the interview, the following information will be provided:

The confidentiality of healthcare personnel, voluntary participation, the opportunity to withdraw at any time, all audio recordings will be deleted upon project completion, and all data will be anonymized.

The questions may evolve during the interview based on the discussions that emerge. The main themes are outlined below along with the corresponding questions.

Focus Group Interview

Questions:

- How did you experience the use of VR simulation?

- What have you learned from observing the demonstration of threatening behaviour through the VR simulation?

- Possible follow-up question: What have you learned about communication skills? Or other follow-up questions related to other themes that arise during the conversation.

- How do you envision applying the experiences gained from the VR simulation in practice?

- What areas do you feel you need to acquire more knowledge about?

- Is there anything you have experienced that we have not asked about?
